# 2190 Longitudinal changes in EEG power envelope connectivity are proportional to motor recovery in chronic stroke patients

**DOI:** 10.1017/cts.2018.92

**Published:** 2018-11-21

**Authors:** Joseph B. Humphries, David T. Bundy, Eric C. Leuthardt, Thy N. Huskey

**Affiliations:** 1 Institute of Clinical and Translational Sciences, Washington University in St. Louis; 2 Post-Doctoral Trainee, University of Kansas Medical Center; 3 Mid-Career Investigator, Washington University School of Medicine in St. Louis

## Abstract

OBJECTIVES/SPECIFIC AIMS: The objective of this study is to determine the degree to which the use of a contralesionally-controlled brain-computer interface for stroke rehabilitation drives change in interhemispheric motor cortical activity. METHODS/STUDY POPULATION: Ten chronic stroke patients were trained in the use of a brain-computer interface device for stroke recovery. Patients perform motor imagery to control the opening and closing of a motorized hand orthosis. This device was sent home with patients for 12 weeks, and patients were asked to use the device 1 hour per day, 5 days per week. The Action Research Arm Test (ARAT) was performed at 2-week intervals to assess motor function improvement. Before the active motor imagery task, patients were asked to quietly rest for 90 seconds before the task to calibrate recording equipment. EEG signals were acquired from 2 electrodes—one each centered over left and right primary motor cortex. Signals were preprocessed with a 60 Hz notch filter for environmental noise and referenced to the common average. Power envelopes for 1 Hz frequency bands (1–30 Hz) were calculated through Gabor wavelet convolution. Correlations between electrodes were then calculated for each frequency envelope on the first and last 5 runs, thus generating one correlation value per subject, per run. The chosen runs approximately correspond to the first and last week of device usage. These correlations were Fisher Z-transformed for comparison. The first and last 5 run correlations were averaged separately to estimate baseline and final correlation values. A difference was then calculated between these averages to determine correlation change for each frequency. The relationship between beta-band correlation changes (13–30 Hz) and the change in ARAT score was determined by calculating a Pearson correlation. RESULTS/ANTICIPATED RESULTS: Beta-band inter-electrode correlations tended to decrease more in patients achieving greater motor recovery (Pearson’s *r*=−0.68, *p*=0.031). A similar but less dramatic effect was observed with alpha-band (8–12 Hz) correlation changes (Pearson’s *r*=−0.42, *p*=0.22). DISCUSSION/SIGNIFICANCE OF IMPACT: The negative correlation between inter-electrode power envelope correlations in the beta frequency band and motor recovery indicates that activity in the motor cortex on each hemisphere may become more independent during recovery. The role of the unaffected hemisphere in stroke recovery is currently under debate; there is conflicting evidence regarding whether it supports or inhibits the lesioned hemisphere. These findings may support the notion of interhemispheric inhibition, as we observe less in common between activity in the 2 hemispheres in patients successfully achieving recovery. Future neuroimaging studies with greater spatial resolution than available with EEG will shed further light on changes in interhemispheric communication that occur during stroke rehabilitation.

